# Targeting p53-Driven FOXM1 Suppresses Tumor Growth and Synergistically Sensitizes to Chemotherapy in Triple-Negative Breast Cancer Models

**DOI:** 10.3390/cells15141237

**Published:** 2026-07-09

**Authors:** Sayra Dilmac, Nermin Kahraman, Ferah Comert Onder, Ogun Ali Gul, Bulent Ozpolat

**Affiliations:** 1 Department of Nanomedicine, Houston Methodist Research Institute, Houston, TX 77030, USA; sdilmac@ou.edu (S.D.); nkahraman@mdanderson.org (N.K.); ogunaligul@gmail.com (O.A.G.); 2 Department of Medical Biology, Faculty of Medicine, Çanakkale Onsekiz Mart University, Çanakkale 17100, Türkiye; ferahcomertonder@comu.edu.tr; 3 Department of Experimental Therapeutics, MD Anderson Cancer Center, The University of Texas, Houston, TX 77054, USA; 4 Stephenson School of Biomedical Engineering, The University of Oklahoma, Norman, OK 73019, USA

**Keywords:** FOXM1, TNBC, small molecule inhibitor, FOXM1 inhibitor

## Abstract

Triple-negative breast cancer (TNBC) is characterized by a lack of estrogen, progesterone, and HER2 receptors; an aggressive phenotype; high rates of early relapse and metastasis; and the worst mortality rates among all breast cancer subtypes. Currently, there is no effective curative targeted therapy for TNBC and chemotherapy remains the primary treatment for TNBC. Therefore, there is a critical need to develop highly effective, novel therapies to improve patient survival. We previously validated FOXM1, a proto-oncogenic transcription factor, for the first time as a potential molecular target in TNBC through genetic knockdown studies in mice. We show that FOXM1 expression is associated with shorter patient survival and is a marker of poor prognosis. There is no FDA-approved FOXM1 inhibitor. We found that patients with *TP53* mutations have dramatically higher FOXM1 expression, indicating that widespread *TP53* mutations detected in about 80% of TNBC patients are the major driver of FOXM1 overexpression in TNBC patients. We identified its binding ability using an *in silico* study, and found it to be a well-known FOXM1 inhibitor that suppresses TNBC cell proliferation, migration, and invasion, and induces apoptosis. *In vivo* studies in mice bearing TNBC tumors demonstrated that treatment with a novel FOXM1 inhibitor incorporated in single-lipid nanoparticles suppressed the growth of TNBC tumor xenografts. In conclusion, our findings suggest that the novel FOXM1 inhibitor represents a potent and safe therapeutic strategy with significant potential for the treatment of other FOXM1-driven cancers including TNBC that currently have limited treatment options.

## 1. Introduction

Breast cancer is the most commonly diagnosed cancer and second leading cause of cancer-related deaths in women. Triple-negative breast cancer (TNBC) accounts for 15–20% of all breast cancer cases and is characterized by the lack of expression of estrogen receptors (ER), progesterone receptors (PR), and human epidermal growth factor receptors (HER2) [1,2,3]. Therefore, TNBC patients do benefit from therapies targeting ER, PR, or HER2 receptors, and chemotherapy remains the main medical treatment for TNBC [4]. Unfortunately, about 50–70% of patients cannot achieve complete pathological remission in response to neoadjuvant chemotherapy [5]. Significant heterogeneity, chemoresistance, and early relapses, along with metastasis to the brain, lungs, and liver, contribute to poor patient survival, with the median overall survival in metastatic cases being about 18 months [3,4].

Although several targeted therapies including chemo drug-conjugated anti-Trop-2 antibody (Sacituzumab govitecan) and immunotherapy (e.g., PD-1 (Programmed cell death protein 1)/PD-L1 (Programmed death ligand 1) check-point inhibitors like pembrolizumab and atezolizumab) have been approved by the Food and Drug Administration (FDA), they are not curative therapies for TNBC [6]. Sacituzumab has demonstrated a survival benefit, with a median overall survival of approximately 12.1 months in metastatic TNBC patients, compared to around 6.7 months with standard chemotherapy. Similarly, immunotherapy with pembrolizumab or atezolizumab, when combined with chemotherapy, has shown improved progression-free survival (PFS) and overall survival (OS) in PD-L1-positive TNBC patients, with a median OS ranging from 16 to 21 months in clinical trials compared to 12 to 15 months for patients receiving chemotherapy alone [7,8].

The *TP53* gene is one of the major tumor suppressor genes that regulates cell cycle progression, DNA repair, cellular senescence, and apoptosis. In all human cancers, *TP53* mutations are the most common single-gene alterations, occurring in about 50% of patients. While *TP53* mutations are detected in 30–35% of all breast cancer cases, their prevalence is significantly higher in TNBC, with an 80% mutation rate—making it one of the most common genetic alterations and a key molecular determinant of TNBC [9]. The presence of the *TP53* mutation in TNBC patients is associated with significantly worse prognosis compared to other subtypes [9]. Therefore, identifying signaling pathways downstream of mutated *TP53* could be an approach for the development of a common treatment for TNBC.

Forkhead box M1 (FOXM1), a member of the Forkhead transcription factor family, is critical, driving the tumorigenesis and progression of aggressive solid cancers such as TNBC, pancreatic, lung, and brain cancer [10,11,12], and is a proto-oncogenic transcription factor that contributes to cell proliferation, invasion tumorigenesis, and the progression of TNBC [11,12,13,14]. FOXM1 plays an essential role in regulating cell cycle phases, including the G1-S, G2, and M phase transitions, mitosis, angiogenesis, oxidative stress, and inflammation [15,16,17,18].

Our previous studies validated FOXM1 as a potential molecular target in TNBC for the first time using genetic knockdown approaches and demonstrated that genetic inhibition of FOXM1 completely blocks TNBC tumor growth in multiple mouse tumor models, with no detected toxicity [10]. Currently, there is no FDA-approved FOXM1 inhibitor.

In the current study, we found that FOXM1 is broadly overexpressed in TNBC patient tumors with *TP53* mutations and is associated with dramatically shorter patient survival. We demonstrated that FOXM1 inhibition by a newly identified inhibitor according to *in silico* analysis suppresses FOXM1. FOXM1 inhibition suppresses cell proliferation, inhibits invasion, induces apoptosis, and synergizes with first-line standard chemotherapeutics. More importantly, in vivo administration of the new inhibitor at low doses (50 mg/kg) suppressed tumor growth in orthotopic TNBC tumor xenografts with no signs of toxicity. Overall, our findings suggest that the novel FOXM1 inhibitor is a potent, in vivo-effective agent and may be used to target FOXM1 in TNBC patients.

## 2. Materials and Methods

### 2.1. Cell Lines and Cell Culture Conditions

The human mammary epithelial cell line MCF-10A, along with the TNBC cell lines MDA-MB-231, MDA-MB-436, BT20, BT549, HCC70, and HCC1937, were obtained from the American Type Culture Collection (ATCC) (Manassas, VA, USA). MCF-10A, MDA-MB-231, MDA-MB-436, and BT20 cells were maintained in DMEM/F12 medium (Corning; #15-090-CV, New York, NY, USA), whereas BT549, HCC70, and HCC1937 cells were grown in RPMI 1640 medium (Gibco; #11875093, Waltham, MA, USA). Both culture media were supplemented with 10% FBS (GenDepot; #F0600-050, Baker, TX, USA) and 1% penicillin–streptomycin solution (Gibco; #15140122, MA, USA). All cell lines were incubated under standard humidified conditions at 37 °C with 5% CO_2_ and 95% air.

### 2.2. Generation of FOXM1-Overexpressing Cells

Lentiviral transduction of MDA-MB-231 cells was performed using a human FOXM1-expressing lentiviral vector (NM_202002.2) driven by the CMV promoter (LPP-U0633-Lv105; GeneCopoeia, Rockville, MD, USA), with a mock lentiviral vector (LPP-NEG-Lv103; GeneCopoeia, MD, USA) serving as the negative control. Transductions were executed following the manufacturer’s instructions, and the upregulation of FOXM1 protein expression was verified by Western blotting.

### 2.3. Clinical Correlation Evaluation of FOXM1 Expression and Patient Survival

Transcriptomic and clinical data from breast cancer patient cohorts were analyzed to evaluate FOXM1 expression patterns across different tissue types, disease stages, and mutational profiles. FOXM1 mRNA expression levels were compared between normal breast tissues and tumor/metastatic tissues, as well as across different pathological stages (Stages I, II, III, and IV).

For survival analyses, a total cohort of 1879 breast cancer patients were stratified into high (*n* = 1311) and low FOXM1 expression groups to determine overall survival and Relapse-Free Survival (RFS) using Kaplan–Meier curves. Additionally, FOXM1 expression was assessed in relation to lymph node metastasis status (*n* = 330). Given the regulatory role of *TP53* in modulating FOXM1 transcription, patient samples were stratified by *TP53* status to compare FOXM1 levels between *TP53*-mutant (*n* = 93) and *TP53*-wild-type (*n* = 124) cohorts. Inter-group statistical comparisons and survival significance were determined using appropriate parametric/non-parametric tests and the log-rank test. The prognostic value of FOXM1 expression in triple-negative breast cancer patients was evaluated using the online database Kaplan–Meier Plotter (kmplot.com). RNA-seq data and clinical parameters were sourced from The Cancer Genome Atlas (TCGA) dataset. Patients were stratified into high and low FOXM1 expression groups based on the median expression level of the gene. Survival curves, including hazard ratios (HR) and log-rank *p*-values, were automatically generated by the platform to determine statistical significance between the two cohorts.

### 2.4. Small Interfering RNA (siRNA) Transfections

MDA-MB-231, MDA-MB-436, and BT20 cells were inoculated into 6-well culture plates at a density of 1.5 × 10^5^ cells/well. Transfections were performed under serum-free conditions using HiPerFect transfection reagent (Qiagen; #301707, Germantown, MD, USA) with either 100 nmol/L FOXM1-targeted siRNA or a non-targeting control siRNA in serum-free DMEM. Following a 6 h incubation period, the transfection medium was replaced with fresh DMEM enriched with 10% FBS. The cells were then maintained under standard incubation conditions for up to 72 h prior to subsequent analyses.

Cells were seeded in a T25 flask, and 24 h after seeding, we treated the cells with 5NT (Cayman, #21517, Ann Arbor, MI, USA) at 1, 2, and 5 μM for WB. Seventy-two hours later, we collected the cells and prepared them for protein extraction.

### 2.5. Protein Extraction and Western Blotting

Following a 72 h transfection period with FOXM1 or control siRNA, MDA-MB-231 and MDA-MB-436 cells were lysed for protein collection. Western blot analyses were carried out as previously detailed [19]. For protein detection, membranes were probed with primary antibodies specific to FOXM1 (Cell Signaling, #5406, Danvers, MA, USA/Santa Cruz, #sc-271746, Santa Cruz, CA, USA) and p53 (Cell Signaling, #2524, MA, USA). Protein bands were visualized using corresponding horseradish peroxidase-conjugated secondary antibodies. To ensure equal protein loading, GAPDH (Cell Signaling, #5174, MA, USA) was used as a reference control. The reproducibility of the data was validated through three independent experimental runs.

### 2.6. Clonogenic Survival Assays and In Vitro Cytotoxicity

To assess long-term survival, MDA-MB-231, MDA-MB-436, BT20, BT549, HCC1395, HCC1806, and HCC70 cells were singularized and deposited into 12-well plates at 300–1000 cells per well. After 48 h of baseline incubation, cells underwent transfection with control siRNA, FOXM1 siRNA, or were subjected to 5NT treatment. Culturing was maintained for 10–14 days until visible colonies appeared. The resulting colonies were visualized via crystal violet staining (Sigma; #C0775, St. Louis, MO, USA) and digitalized for counting through ImageJ software (Version 1.54i, NIH, Bethesda, MD, USA). Data reflect three independent biological replicates performed in triplicate.

Short-term cell viability and proliferation were monitored via the MTS colorimetric method [19]. MDA-MB-231 and MDA-MB-436 cells (1–2 × 10^3^ cells/well) were plated in 96-well formats. Following overnight attachment, cells were exposed to the FOXM1 inhibitor FDI-6 (Sigma; #5.33259, MO, USA). At 72 h post-treatment, cells were incubated with MTS solution (Promega; #G1112, Madison, WI, USA). The optical density of each well was subsequently recorded at 490 nm using a Vmax kinetic ELISA microplate reader (Biotek Synergy Neo2, Bellevue, WA, USA).

### 2.7. In Vitro Motility, Migration, and Transwell Invasion Assays

MDA-MB-231, MDA-MB-436, and BT20 cells were established in 6-well formats (1 × 10^5^ cells/well) for 24 h before undergoing transfection with control siRNA or FOXM1 siRNA, or treatment with 5NT. A uniform wound was mechanically created in the cell layer utilizing a sterile 200 µL pipette tip. Following a gentle wash, fresh medium was added. Microscopic validation of the wound area was performed at 0, 24, and 48 h using a Nikon Eclipse (#TE-200-U, Melville, NY, USA) phase-contrast microscope. The rate of migration was analyzed via ImageJ software, with data normalized against the baseline control area at hour 0.

For three-dimensional migration and invasion profiles, 8 µm pore Transwell inserts (Corning #353097, NY, USA) were utilized. Cells previously transfected for 24 h with the respective siRNAs or 5NT were collected. To measure migration, cells were placed into the upper compartment of the Transwell. To monitor invasion, the inserts were modified by pre-coating with Matrigel matrix (Corning, #354234, NY, USA). In both experimental variations, cells were seeded in the top-chamber inserts in a serum-free environment, while the lower compartment received 10% FBS-supplemented medium to drive chemotaxis. After 24 h of incubation, cells on the lower surface of the membrane were fixed and visualized with Hema 3 stain (Thermo Scientific; #22-122911, Waltham, MA, USA). The degree of migration or invasion was determined by averaging cell counts from five distinct fields under light microscopy. Experimental replicates were performed in triplicate.

### 2.8. Evaluation of Apoptosis

Apoptosis was assessed by annexin V/Propidium iodide staining and flow cytometry analysis. MDA-MB-231, MDA-MB-436, and BT20 cells were seeded in 6-well plates and treated with 5NT (2.5, 5, and 10 μM) and then analyzed using annexin V/propidium iodide staining according to the manufacturer’s protocol (FITC–Annexin V kit; BD Pharmingen; #556547, San Diego, CA, USA). Fluorescence-activated cell sorting analysis (FACS) detected and quantified the positive cells.

### 2.9. Detection of Synergism

To detect the synergistic effect of 5NT with chemotherapeutics such as doxorubicin, we seeded cells in 96-well plates, as in the MTS assay. Twenty-four hours later, we treated cells with 5NT (for MDA-MB-231 cells: 4000, 2000, 1000, 500, and 250 nM; for MDA-MB-436 cells: 6000, 3000, 1500, 750, and 375 nM) for 48 h. Forty-eight hours later, we treated cells with doxorubicin (1000, 500, 250, 125, 60, 30, 15, 7.5, and 5 nM) for 24 h. MTS dye was added 72 h later, and the cells and plates were analyzed on a Vmax kinetic ELISA microplate reader (Molecular Devices, San Jose, CA, USA) and read at 490 nm wavelength. Drug combination synergy was quantified using the Bliss Independence reference model via the SynergyFinder database (https://synergyfinder.fimm.fi, accessed on 30 March 2024). Statistical analysis and score deviation calculations were automatically performed based on three independent biological replicates (*n* = 3).

### 2.10. In Vivo Tumor Xenograft Model of TNBC

Athymic Nu/Nu female mice (6 weeks old) were obtained from Taconic Biosciences (Waltham, MA, USA). All animal studies were conducted according to an experimental protocol approved by the Houston Methodist Research Institute Institutional Animal Care and Use Committee (protocol code IS00008642 and date of approval 12 September 2024). MDA-MB-231 cells (2 × 10^6^ in 30% Matrigel) were injected into the mammary fat pad of each mouse. Seven days after injection, when tumors reached 3–5 mm in size, liposomal siRNA treatment (i.p. to i.v.) was initiated (*n* = 5 per group). Each mouse received 50 mg/kg 5NT incorporated into liposomes three times a week for four weeks, and tumor volumes were measured weekly using a caliper.

### 2.11. In Silico Study

The molecular docking study was conducted according to our previously reported studies [19,20,21]. AutoDock tools (version 1.5.7) were used to prepare the ligand and reference inhibitor FDI-6. Ligand 5NT was drawn using ChemDraw (version 18.0) and saved in sdf format using Chem3D (version 18.0). The structure of FDI-6 was downloaded from PubChem in pdf format. Then, the structures of the ligands were converted into PDBQT format for the molecular docking analysis. The validation of molecular docking analysis was performed using reference inhibitor FDI-6. The crystal structure of FOXM1 (PDB ID: 3G73) was retrieved from the Protein Data Bank (PDB) (https://www.rcsb.org/) and chain B was selected to conduct the analysis. To prepare the protein, polar hydrogens were added, and the processed structure was saved in PDBQT format. A grid box was generated using AutoDock Tools and the binding site residues, including His287 of the FOXM1 DNA-binding domain (DBD), were identified to define the docking protocol. Then, a molecular docking study was executed using the graphical tools AMDock (version 1.6.1) and AutoDock Vina (version 1.1.2) [22]. BIOVIA Discovery Studio (DS 2022) was used to visualize protein–ligand interactions.

### 2.12. Statistical Analysis

Quantitative data are presented as the mean ± standard deviation (SD). Statistical comparisons between the control and multiple treatment groups were evaluated using one-way analysis of variance (ANOVA), while differences between two distinct groups were analyzed using the two-tailed Student’s *t*-test. A *p*-value of <0.05 was designated as the threshold for statistical significance. All statistical computations were executed using GraphPad Prism software (version 6.02). For patient cohort analyses, individuals were stratified into distinct percentiles based on their respective mRNA or miRNA expression profiles. The correlation between target expression levels and overall survival was evaluated via the log-rank test, and cumulative survival distributions were visualized using the Kaplan–Meier method.

## 3. Results

### 3.1. FOXM1 Expression Is Associated with Poor Patient Survival in Breast Cancer Patients

**Figure 1:**
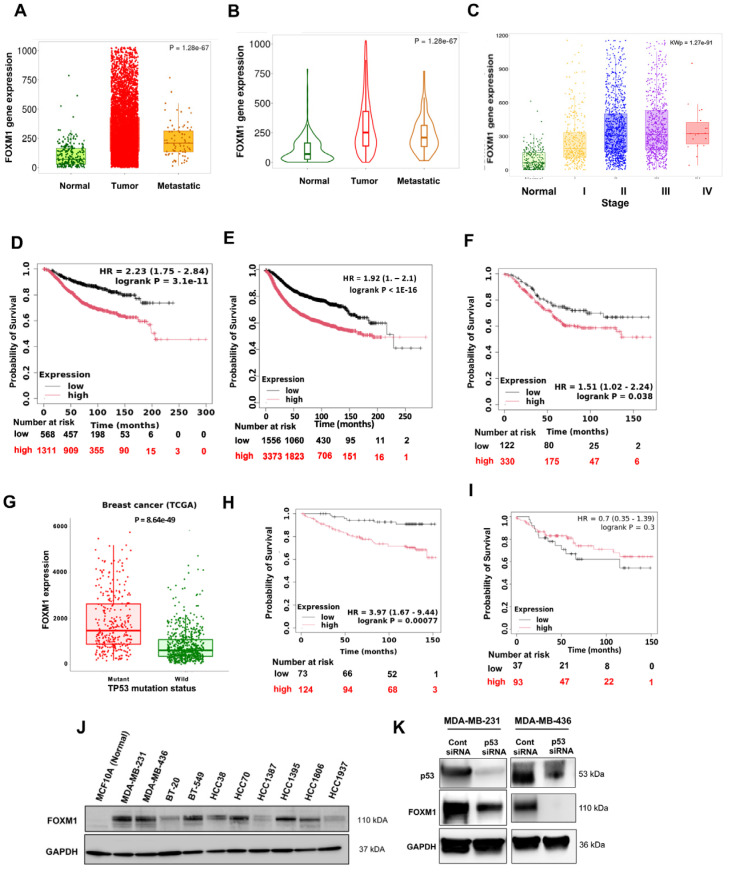
(**A**,**B**) FOXM1 gene expression in normal breast tissues and primary and metastatic breast cancer samples. (**C**) FOXM1 gene expression in patients across different stages of breast cancer (TCGA patient database). (**D**–**F**) High FOXM1 expression is significantly associated with poor overall survival (OS) (**D**) and Relapse-Free Survival (RFS) (**E**) in all breast cancer patients, including lymph node-positive breast cancer patients (**F**) as indicated by Kaplan–Meier survival analysis. (**G**) FOXM1 is overexpressed in TNBC patient tumors harboring *TP53* mutations and associated with shorter overall survival in breast cancer patients with *TP53* mutations (**H**) but not WT *TP53* (**I**). (**J**) FOXM1 protein expression is upregulated in TNBC cell lines compared to normal breast epithelial cells (MCF-10A) according to Western blot analysis. (**K**) Knockdown of mutated FOXM1 by siRNA leads to inhibition of FOXM1 in MDA-MB-231 and MDA-MB-436 TNBC cell lines harboring mutant *TP53* mutations) detected by Western blot analysis.

To elucidate the clinical significance of FOXM1 expression, we analyzed all breast cancer patients using the TCGA database. A Kaplan–Meier survival analysis was performed to assess its role in patient survival and prognosis. A comparison of normal, tumor, and metastatic tissues showed that FOXM1 gene expression was higher in tumor and metastatic tissues than in normal tissues (Figure 1A,B). FOXM1 gene expression was higher in stage II and III tumors than in stage I and IV tumors and in normal tissue (Figure 1C). Among all of the breast cancer patients (*n* = 1879), 1311 had high FOXM1 expression (Figure 1D). The RFS (Relapse-Free Survival) analysis also revealed that FOXM1 expression was high (Figure 1E) in TNBC patients with lymph node metastasis (*n* = 330) (Figure 1F). Considering that *TP53* is responsible for regulating FOXM1 expression, Kaplan–Meier analyses showed that FOXM1 expression was higher in the *TP53* mutation compared to the wild type (Figure 1G). Similarly, FOXM1 expression was often high in breast cancer patients with mutant *TP53* (*n* = 93) (Figure 1I), but not in wild-type *TP53* patients (*n* = 124) (Figure 1H).

### 3.2. TP53 Mutations Drive FOXM1 Overexpression in TNBC Cell Lines

To evaluate the baseline expression of FOXM1, we initially screened a panel of triple-negative breast cancer (TNBC) cell lines alongside the non-transformed human mammary epithelial cell line, MCF-10A, via Western blot analysis. The results demonstrated that FOXM1 protein levels were significantly elevated across all tested TNBC cell lines compared to the MCF-10A control cells (Figure 1J). When p53 expression was reduced in MDA-MB-231 and MDA-MB-436, FOXM1 decreased similarly with p53 siRNA (Figure 1K).

### 3.3. FOXM1 Drives TNBC Cell Proliferation

**Figure 2:**
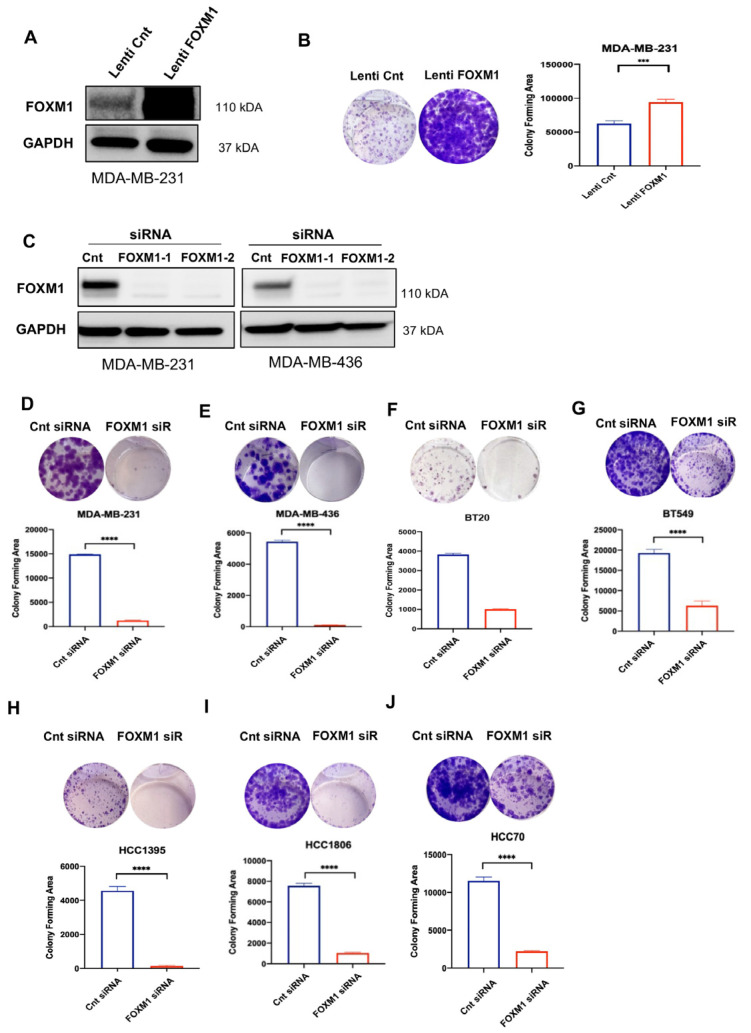
(**A**) FOXM1 protein expression is high in MDA-MB-231 cells transduced with FOXM1 lentivirus. (**B**) FOXM1-overexpressing MDA-MB-231 cells exhibit increased colony formation compared with control cells. (**C**) FOXM1 inhibition with FOXM1 siRNA is demonstrated in MDA-MB-231 and MDA-MB-436 cells. (**D**–**J**) Effect of FOXM1 inhibition with FOXM1 siRNA on MDA-MB-231, MDA-MB-436, BT20, BT549, HCC1395, HCC1806, and HCC70 cells reduces colony formation (*** *p* < 0.0005, **** *p* < 0.0001).

We transfected cells with FOXM1 lentivirus and showed that the FOXM1 protein expression level (Figure 2A) induces cell proliferation and colony formation (Figure 2B). We also found that the inhibition of FOXM1 with two different siRNAs (100 nM) suppressed FOXM1 protein expression in MDA-MB-231 and MDA-MB-436 (Figure 2C) compared with control siRNA. We also investigated the role of FOXM1 in the MDA-MB-231, MDA-MB-436, BT20, BT549, HCC1395, HCC1806, and HCC70 breast cancer cell lines. Our results showed that FOXM1 promotes cell proliferation and colony formation (Figure 2D–J). Notably, silencing FOXM1 using siRNA significantly reduced colony formation in these cell lines.

### 3.4. FOXM1 Promotes Cell Migration and Invasion of TNBC Cells

**Figure 3:**
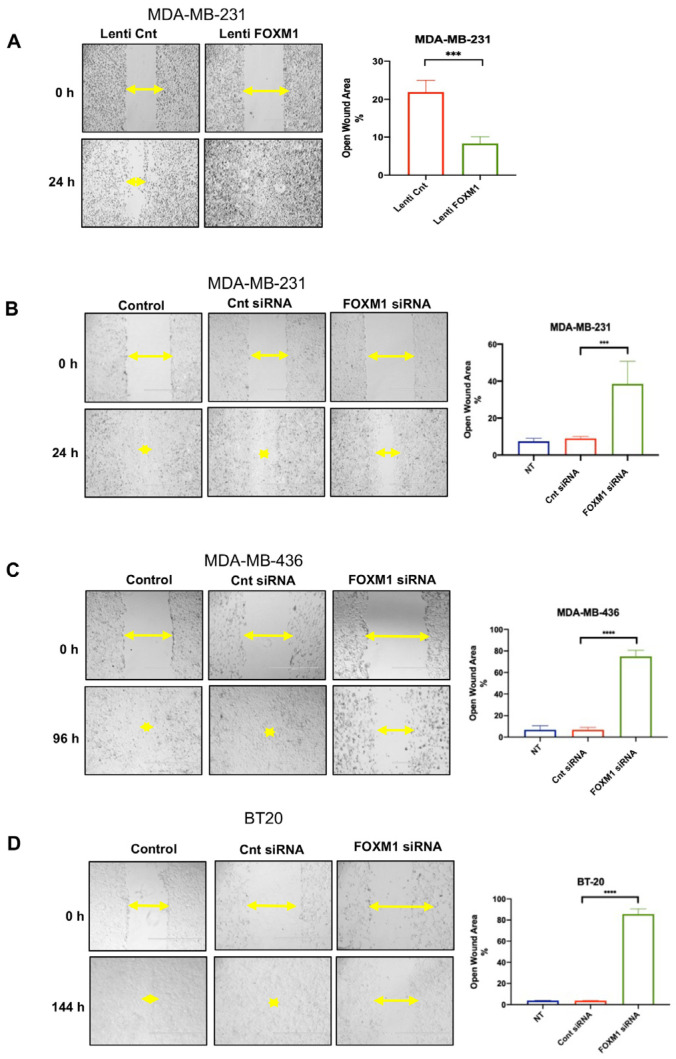
(**A**) FOXM1 overexpression promotes migration in MDA-MB-231 cells. FOXM1 inhibition with FOXM1 siRNA reduces migration on MDA-MB-231 (**B**), MDA-MB-436 (**C**), and BT20 (**D**) cells (*** *p* < 0.0005, **** *p* < 0.0001) (The yellow arrows represent the wound distance).

**Figure 4:**
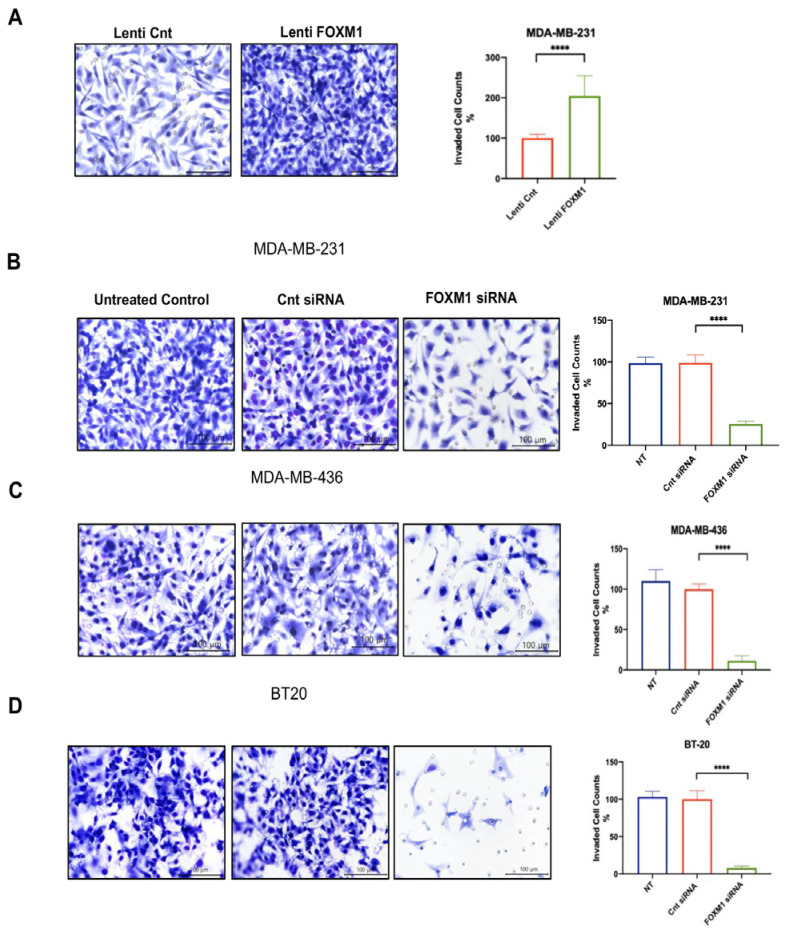
(**A**) Invasion capability increased in MDA-MB-231 cells transduced with lentivirus expressing FOXM1. FOXM1 inhibition using FOXM1 siRNA reduced the invasion potential of MDA-MB-231 (**B**), MDA-MB-436 (**C**), and BT20 (**D**) cells (**** *p* < 0.0001).

To demonstrate the role of FOXM1 in promoting cell motility and invasion, we overexpressed and knocked down FOXM1 expression using a lentivirus and siRNA, respectively. The migratory and invasive capacities of MDA-MB-231, MDA-MB-436, and BT20 cells were evaluated after transfection with FOXM1 siRNA, and MDA-MB-231 cells transfected with FOXM1 lentivirus were subjected to migration and invasion assays using Transwell invasion chambers. MDA-MB-231 cells transfected with FOXM1 lentivirus showed higher invasion and migration potential than MDA-MB-231 cells with control lentivirus (Figure 3A and Figure 4A). Treatment with FOXM1 siRNA reduced invasion and migration in MDA-MB-231 (Figure 3B and Figure 4B), MDA-MB-436 (Figure 3C and Figure 4C), and BT20 (Figure 3D and Figure 4D) cells compared with controls.

### 3.5. Molecular Docking Analysis

**Figure 5:**
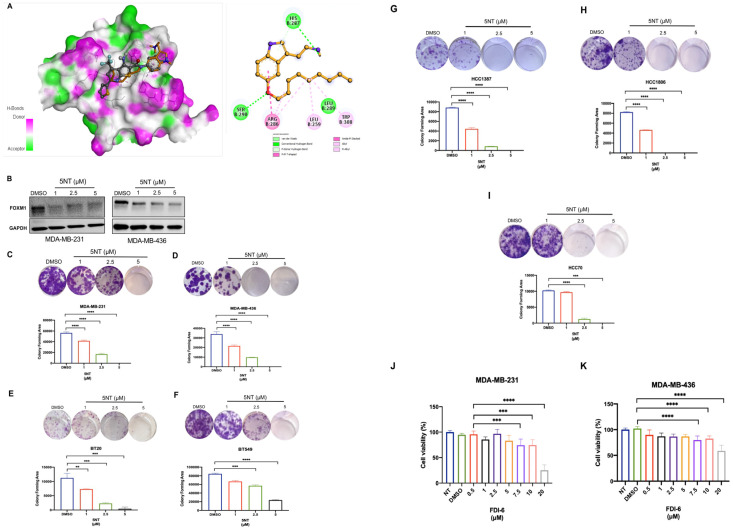
(**A**) Molecular docking for compound 5NT (orange). 3D surface representation and 2D ligand interaction diagram of 5NT in the FOXM1-DBD binding pocket overlapping with FOXM1 reference inhibitor FDI-6 (dark grey). (**B**) 5NT treatment reduces FOXM1 expression in MDA-MB-231 and MDA-MB-436 cells at 1 μM and higher concentrations. (**C**–**I**) 5NT also suppresses colony formation in MDA-MB-231, MDA-MB-436, BT20, and BT549 cells. FDI-6 exhibits an IC_50_ of ~10 μM in MDA-MB-231 (**J**) and MDA-MB-436 (**K**) cells (** *p* < 0.005, *** *p* < 0.0005, **** *p* < 0.0001).

A molecular docking study was performed to investigate the binding interactions of compound 5NT with the FOXM1 DNA-binding domain (FOXM1-DBD, PDB: 3G73). This *in silico* analysis aimed to evaluate the target critical binding sites, yielding a calculated binding ability of −4.9 kcal/mol. A pivotal finding of the study highlights the role of the evolutionarily conserved residues Asn283, Arg286, and His287, which are indispensable for the stabilization of the protein-DNA complex [20,23,24,25]. The molecular docking study showed that the crucial interactions, including His287 and Arg286, were observed with compound 5NT in the FOXM1-DBD (Figure 5A). The stability of this complex is further enhanced by a hydrogen bonding network established through His287 and Ser290, while Arg286, Leu256, and Trp308 engage in specialized non-covalent contacts, including pi–pi T-shaped, hydrophobic pi–alkyl, and alkyl interactions. A comparative analysis with the reference inhibitor FDI-6 suggested that 5NT has binding capacity for FOXM1-DBD.

In this study, the binding energy of FDI-6 was calculated as −5.8 kcal/mol. Furthermore, we have previously reported the binding score and interaction analysis of FDI-6 [19,20]. The indole ring contributed to the formation of hydrogen bond and pi–pi interactions, respectively, with the His287 and Arg286 residues. Conventional hydrogen bonds occurred between an oxygen atom and Ser290, and between the hydrogen atom of an aliphatic amino group and His287. Additionally, CH_2_ groups in the alkyl chain, linked to the phenyl ring by an ether bridge, interact with residues Trp308, Leu259, and Arg286 to form alkyl and pi–alkyl interactions (Figure 5A).

As a result, the *in silico* analysis indicates that the molecular architecture of 5NT enables favorable interactions with functionally important residues in FOXM1-DBD. These structural insights may guide the rational optimization of FOXM1 inhibitors with improved therapeutic potential.

### 3.6. A Novel Inhibitor Suppresses FOXM1 Expression and Cell Proliferation

We treated MDA-MB-231 and MDA-MB-436 cells with 5NT at 1, 2.5, and 5 μM. We found that 5NT inhibits FOXM1 expression at 1 μM and at higher doses (Figure 5B).

We performed a clonogenic assay to evaluate the effects of 5NT on TNBC cell proliferation. We found that treatment of TNBC cells with 5NT inhibited colony formation in MDA-MB-231, MDA-MB-436, BT20, BT549, HCC1387, HCC1806, and HCC70 cells at 1 μM and higher doses (Figure 5C–I). Furthermore, we used FDI-6, an inhibitor of FOXM1, as a positive control and also compared the effects with the novel inhibitor. FDI-6 binds directly to the FOXM1 protein and inhibits FOXM1. We performed an MTS assay to detect cell viability with FDI-6. We found that the FDI-6 IC50 is higher at 10 μM in both the MDA-MB-231 and MDA-MB-436 cell lines (Figure 5J,K).

### 3.7. Inhibition of FOXM1 Inhibits Cell Migration and Invasion of TNBC

**Figure 6:**
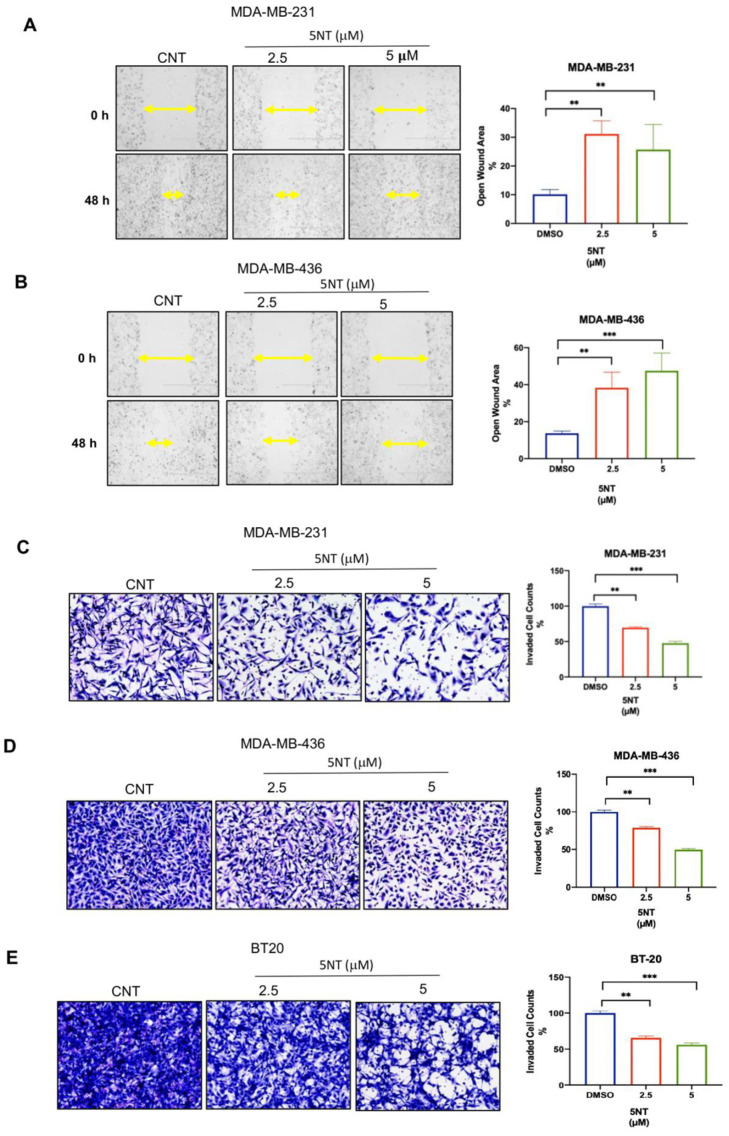
5NT inhibits cell migration for MDA-MB-231 (**A**) and MDA-MB-436 (**B**) cells. 5NT reduces invasion potential of MDA-MB-231 (**C**), MDA-MB-436 (**D**), and BT20 (**E**) cell lines (** *p* < 0.005, *** *p* < 0.0005).

Based on the finding that FOXM1 siRNA inhibited FOXM1 and affected the migration and invasion of TNBC cells, we wanted to determine whether 5NT similarly affected TNBC cell migration and invasion. MDA-MB-231 and MDA-MB-436 cells were treated with 5NT at 2.5 and 5 μM. After 48 h of 5NT treatment control, the wound area was closed in the MDA-MB-231 and MDA-MB-436 cells (Figure 6A,B). On the contrary, MDA-MB-231 and MDA-MB-436 cells treated with 5NT showed protection against the wound at 2.5 and 5 μM doses (Figure 6A,B). Also, MDA-MB-231, MDA-MB-436, and BT20 cells treated with 5NT slowly traversed into the Matrigel, and the migrated cell count was statistically reduced compared to the control group (Figure 6C–E).

**Figure 7:**
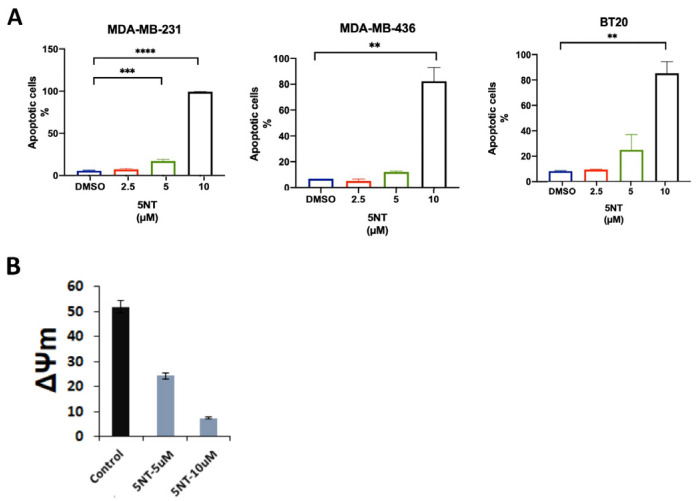
(**A**) FOXM1 inhibitor 5NT induces apoptosis in TNBC (** *p* < 0.005, *** *p* < 0.0005, **** *p* < 0.0001). (**B**) Mitochondrial membrane potential decreases in a dose-dependent manner in MDA-MB-231 cells following 24 h of treatment with 5NT.

We used 5NT to detect apoptosis in MDA-MB-231, MDA-MB-436, and BT20 cells at 2.5, 5, and 10 μM. Our results showed that 5NT induced apoptosis at 5 μM and higher doses in MDA-MB-231 cells (Figure 7A). Also, in MDA-MB-436 and BT20 cells, 5NT induced apoptosis at 10 μM (Figure 7A).

The mitochondrial membrane potential was measured after MDA-MB-231 cells were treated with 5 µM and 10 µM 5NT for 24 h. A dose-dependent decrease in mitochondrial membrane potential was observed compared to the control (Figure 7B).

### 3.8. 5NT Synergizes with Standard Chemotherapeutics (Doxorubicin) in TNBC

**Figure 8:**
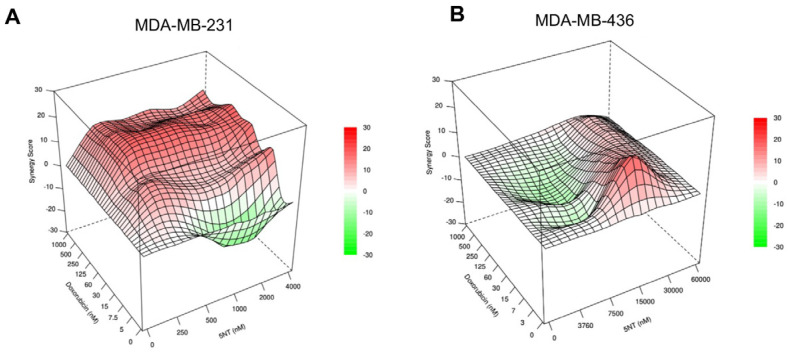
5NT and doxorubicin synergize on MDA-MB-231 (**A**) and MDA-MB-436 (**B**) cell lines.

We performed a synergism assay to determine the combined effect of 5NT and doxorubicin. Our results showed that a combination of 5NT and doxorubicin exhibits strong synergism when used together. In MDA-MB-231, the 5NT and doxorubicin combination synergism score is 11.504; in MDA-MB-436, the synergism score is 1.814 (Figure 8A,B). This result showed us that combining 5NT and doxorubicin could be a more robust treatment method for TNBC.

### 3.9. Therapeutic Targeting of FOXM1 by 5NT Inhibits the In Vivo Tumor Growth of Orthotopic TNBC Xenograft Tumors in Mice

**Figure 9:**
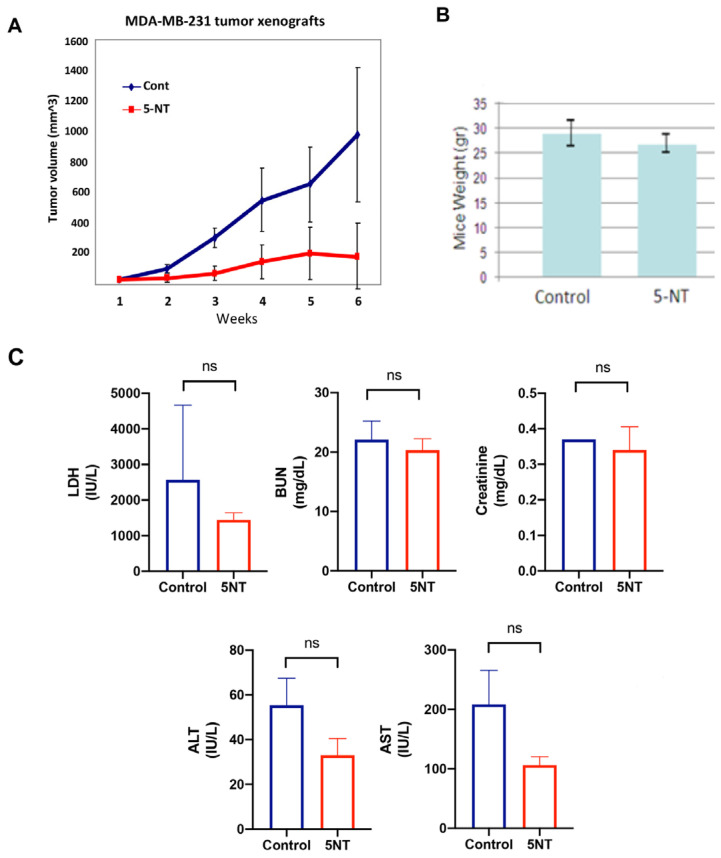
(**A**) 5NT inhibits tumor growth in an orthotopic mouse MDA-MB-231 xenograft model. (**B**) Mouse body weights remained unchanged following 5NT treatment, indicating no detectable toxicity. (**C**) Serum biochemistry analysis of 5NT-treated mice. Serum levels of ALT, AST, BUN, Creatinine, and LDH were measured to evaluate systemic toxicity. No significant differences were observed between the control and 5NT-treated groups. Data are presented as mean ± SD (ns: non-significant).

We aimed to determine the *in vivo* role of FOXM1 inhibition with 5NT in an orthotopic xenograft model of MDA-MB-231 in mice. MDA-MB-231 cells were implanted into the mammary fat pad of nude mice by subcutaneous injection, and about seven days later, 5NT (50 mg/kg) in DMPC-based nanoliposomes was injected into mice three times a week for four weeks. Mice treated with liposomal 5NT had significantly smaller tumors than those in the control group (Figure 9A). These results showed that 5NT is an effective and safe treatment for TNBC, with no observed toxicity, as indicated by no change in the mice’s weight (Figure 9B). To evaluate the systemic safety profile of 5NT, comprehensive serum chemistry analyses were conducted at the conclusion of the xenograft study. Animals treated with 5NT exhibited normal levels of hepatic biomarkers (ALT and AST) and renal biomarkers (BUN and Creatinine), indicating no treatment-related liver or kidney toxicity. Additionally, serum LDH levels remained unaltered between the control and treated groups, confirming excellent systemic tolerability (Figure 9C).

## 4. Discussion

FOXM1 is an emerging clinically significant molecular target that is frequently overexpressed and associated with shorter patient survival and poor prognosis in breast cancer. FOXM1 is involved in almost all hallmarks of cancer and drug resistance and promotes tumor growth and progression in TNBC [14,26]. Considering that about a half of TNBC patients are resistant to chemotherapy and the limited benefit of targeted therapeutic options including immunotherapy, new and curative treatment options are needed for treating primary and advanced and metastatic TNBC [27]. Therefore, identifying novel and effective clinically applicable FOXM1 inhibitors may provide a novel treatment alternative for TNBC and other FOXM1-driven solid tumors.

TNBC patient tumors commonly harbor *TP53* mutations, being detected in about 80% of TNBC patients [9]. We found that FOXM1 expression was significantly associated with *TP53* mutations in patients, suggesting that *TP53* mutations contribute to FOXM1 overexpression. In fact, our studies showed that wild-type *TP53* suppresses FOXM1 expression while mutated *TP53* induces FOXM1 expression, as targeting mutated *TP53* led to a reduction in FOXM1 expression, explaining why patients with *TP53* have higher expression of FOXM1. In search of novel FOXM1 inhibitors, our *in silico* analysis identified a potential FOXM1 inhibitor which is significantly more potent than the well-known FOXM1 inhibitor FDI-6. While the *in silico* analysis highlights a favorable structural affinity toward the FOXM1-DBD, this direct physical binding alone cannot completely account for the robust decrease in total FOXM1 expression levels observed in both the TNBC cells and animal models. Our *in vitro* and *in vivo* findings demonstrate that 5NT successfully targets and strongly downregulates FOXM1 expression across dynamic biological systems. We found that the new inhibitor demonstrated significant inhibition of FOXM1 protein expression at a dose as low as 1 μM in TNBC cells. FOXM1 inhibition by siRNA or the new inhibitor similarly reduced cell proliferation and colony formation in various TNBC cell lines (MDA-MB-231, MDA-MB-436, BT20, BT549, HCC1387, HCC1806, and HCC70). We also observed that migration and invasion of TNBC cells was significantly and similarly inhibited by the FOXM1 inhibitor and siRNA.

Compared to classic inhibitors like FDI-6, which functions by interfering with FOXM1 transcriptional activity [28], and Thiostrepton, a thiazole antibiotic known to inhibit FOXM1 proteasomal degradation but limited by poor bioavailability [29], 5NT demonstrates significant functional suppression of FOXM1 signaling at micromolar concentrations. Consequently, 5NT may overcome some of the translational limitations faced by these earlier compounds, presenting a viable alternative for targeted breast cancer therapies [28,29].

While the role of FOXM1 in driving TNBC cell proliferation is well established, its precise contribution to the orchestration of the metastatic cascade remains a critical area of investigation [18]. In this study, we evaluated cell migration and invasion based on the rationale that FOXM1 functions as a pivotal transcriptional driver of the Epithelial–Mesenchymal Transition (EMT). Mechanistically, FOXM1 overexpression has been shown to directly upregulate mesenchymal markers and promote extracellular matrix degradation, largely through the transcriptional activation of downstream effectors such as Snail and Matrix Metalloproteinases (MMPs) [30,31,32]. Our functional assays substantiate this conceptual link, demonstrating that FOXM1 modulation directly impacts the critical phenotypes that govern TNBC cell motility and invasiveness.

More importantly, given the clinical resistance of TNBC patients to chemotherapeutics and the contribution of FOXM1 to chemoresistance, we evaluated the effect of the most commonly used chemo agent doxorubicin (Adriamycin) on FOXM1 and found that doxorubicin dramatically induces FOXM1 expression in TNBC cells, indicating that induced FOXM1 expression may prevent a clinical response to chemotherapeutics and contribute to poor response and shorter patient survival [16,33]. More importantly, FOXM1 inhibition synergistically enhanced the response to doxorubicin and enhanced apoptosis induced by doxorubicin. This case demonstrates that 5NT and doxorubicin exhibit synergistic effects. This led us to believe that combining FOXM1 inhibitor and doxorubicin in the clinic would be a more effective treatment for TNBC.

While our novel FOXM1 inhibitor suppressed tumor growth in the orthotopic TNBC mice model, with toxic effects in mice suggesting that it has a potential for clinical translation, it may only be tested in Phase I clinical trials after more stringent toxicology studies. In conclusion, the new FOXM1 inhibitor is a potential candidate for clinical translation and it may be used alone and in combination with conventional chemotherapeutics for treatment for TNBC and other solid tumors as a FOXM1 inhibitor.

## 5. Conclusions

In conclusion, our findings indicate that *TP53* mutations lead to FOXM1 overexpression in TNBC patients, providing a mechanistic rationale for targeting FOXM1 in *TP53*-mutant TNBC. Importantly, we identify a novel, highly potent inhibitor that effectively targets FOXM1 and suppresses TNBC proliferation, colony formation, migration, invasion, and tumor growth in TNBC xenografts models in mice with no observed toxicity. Moreover, FOXM1 inhibition synergistically enhances chemotherapeutic efficacy and this provides a strong rationale for combination therapy. Collectively, these results highlight the novel FOXM1 inhibitor as a promising therapeutic strategy and support its further preclinical development alone or in combination with standard chemotherapy for the treatment of TNBC and other FOXM1-driven solid tumors.

## Data Availability

The data presented in this study are available on request from the corresponding author due to institutional and ethical restrictions on data sharing.

## Abbreviations

The following abbreviations are used in this manuscript:

ANOVA	One-way analysis of variance
DBD	DNA-binding domain
DS	Discovery Studio
ER	Estrogen receptors
EMT	Epithelial–mesenchymal transition
FDA	Food and Drug Administration
FOXM1	Forkhead box M1
HER2	Human epidermal growth factor receptors
HR	Hazard ratio
MD	Molecular dynamics
MMP	Matrix metalloproteinase
MTS	3-(4,5-dimethylthiazol-2-yl)-5-(3-carboxymethoxyphenyl)-2-(4-sulfophenyl)-2H-tetrazolium
OS	Overall survival
PD1	Programmed cell death protein 1
PD-L1	Programmed death ligand 1
PFS	Progression-free survival
PR	Progesterone receptor
RFS	Relapse-Free Survival
RMSD	Root mean square deviation
TCGA	The Cancer Genome Atlas
TNBC	Triple-negative breast cancer
WB	Western blot
